# 冷冻干燥技术在环境水样有机新污染物前处理中的应用进展

**DOI:** 10.3724/SP.J.1123.2021.02034

**Published:** 2021-08-08

**Authors:** Yiqing ZHANG, Shanshan GUO, Qian SUN

**Affiliations:** 1.中国科学院城市环境研究所, 中国科学院城市污染物转化重点实验室, 福建 厦门 361021; 1. Institute of Urban Environment, Chinese Academy of Sciences, CAS Key Laboratory of Urban Pollutant Conversion, Xiamen 361021, China; 2.中国科学院大学, 北京 100049; 2. University of Chinese Academy of Sciences, Beijing 100049, China; 3.福建农林大学, 福建 福州 350028; 3. Fujian Agriculture and Forestry University, Fuzhou 350028, China

**Keywords:** 前处理方法, 有机新污染物, 冷冻干燥, 环境样品, 综述, pretreatments, emerging organic contaminants, lyophilization, environmental samples, review

## Abstract

有机新污染物是一类在先进分析技术帮助下新鉴定的、现有法规未管制的、人为源的有机污染物。有机新污染物主要包括药品与个人护理、农药、全氟化合物、内分泌干扰物等,其会产生内分泌干扰效应、诱发抗性基因传播,还对人类和野生生物的生存与发展构成潜在威胁,因此检测环境样品中的有机新污染物浓度对生态环境和人体健康具有重大意义。由于环境样品中的有机新污染物浓度较低,为了达到检测仪器的检测要求,通常需要对环境样品进行前处理,包括样品的净化和浓缩。冷冻干燥技术是一种在真空干燥条件下通过升华方式去除水分的前处理技术,主要包括样品冷冻、初级干燥和再干燥3个阶段,常用于食品和药品行业。在药品行业中,冷冻干燥技术能维持药品的生物活性和化学活性,保持药品的物理化学特性。近年来,冷冻干燥技术逐步用于环境水样中有机新污染物的前处理。其主要的操作步骤包括水样预处理、冷冻干燥、洗脱、吹干、过滤、定容和上机检测。冷冻干燥技术具有操作简单、低成本、样品处理体积少、样品易保存和处理过程中样品损失少等优点,具有广泛应用于环境样品中有机新污染物监测的潜力。该文综述了环境样品中有机新污染物常见的种类,并重点介绍冷冻干燥技术的原理及其在环境样品前处理过程中的应用,提出了冷冻干燥技术在环境分析中的应用前景,为环境样品中有机新污染物的监测提供了参考。

## Abstract (en)

With rapid urbanization, increasing amounts of chemicals are being used in our daily life. Emerging organic contaminants are a large class of chemicals that are widely detected in the environment. They include pharmaceuticals and personal care products, pesticides, perfluorinated compounds, and endocrine-disrupting compounds. Recently, many countries have reported the occurrence of emerging organic contaminants. In addition, emerging organic contaminants are frequently detected in various environmental samples, including river and lake water samples. Despite their low concentrations in the environment, emerging organic contaminants are potential risks to humans and wildlife. Because they could lead to endocrine disrupting effects and the occurrence of resistance genes. Thus, the detection of emerging organic contaminants in the environment is imperative for ecological systems and human health. The recent development of analytical techniques has led to the identification of more emerging organic contaminants in the environment. At present, the commonly used chromatographic separation techniques include liquid chromatography and gas chromatography. For the identification and quantitation of emerging organic contaminants, chromatography is usually combined with spectroscopy or mass spectrometry. The concentrations of emerging organic contaminants are not sufficiently high to be detected directly, and the matrix is complex in environmental samples. Emerging organic contaminants also have diverse properties. Thus, environmental samples must be pretreated before detection. Pretreatment includes the concentration and purification of the environmental samples. The commonly used pretreatment methods are ultrasound-assisted extraction, QuEChERS, liquid-liquid extraction, and solid-phase extraction. With the development of pretreatment methods, solid-phase microextraction and stirred-bar adsorption extraction have also become popular. These sample pretreatment methods have many advantages such as good efficiency and effectivity for recycling target compounds. Hence, they are widely used. However, these methods are time-consuming, in addition to requiring expensive consumables and large amounts of organic solvents. Lyophilization is a technique used for product dehydration, preservation, and storage in the agricultural and food industries. It is also occasionally used to simplify the extraction procedure for drug residue analysis. There are three stages of lyophilization: freezing, primary drying, and secondary drying. Lyophilization has many advantages when used for pretreatment, namely, operational simplicity, less consumables, less sample volume, prolonged storage, and minimal sample loss. Lyophilization is thus an alternative method for the pretreatment of emerging organic contaminants in environmental samples. Recently, lyophilization has been applied to the pretreatment of emerging organic contaminants in environmental water samples. In general, a few steps were included during sample analysis. First, the samples were pretreated and frozen before the primary drying stage. After lyophilization, the analytes were extracted using a small amount of organic solvent and dried with nitrogen. Consequently, the elution was reconstructed and detected after dryness and filtration. Many groups of emerging organic contaminants could be recovered, including antibiotics, pesticides, and endocrine-disrupting compounds. The lyophilization could be automated, so that minimal manual intervention was required. By adopting lyophilization, good recoveries and accuracies were achieved. A few cleanup processes were conducted, and a small amount of organic solvent was consumed. Thus, lyophilization is a highly prospective pretreatment method for monitoring emerging organic contaminants, and can be extended to a diverse range of these contaminants. For large-scale and high-frequency sampling campaigns, cheap and convenient pretreatment is urgently needed. In this study, typical emerging organic contaminants are described, along with the universal types of such contaminants and the principles of lyophilization. The application of lyophilization to the detection of emerging organic contaminants in the environment is also introduced. The future of lyophilization is discussed, which provides a reference for emerging contaminant detection in environmental samples.

随着城镇化进程的加快和现代检测技术的发展,越来越多用于提高人民生活水平的化学药品被高灵敏度、高精度的仪器频繁检出。新污染物(contaminant of emerging concerns, CECs)是从环境中新检出或是由人们新合成、未受管制的、人为源的痕量污染物,其中大多数为有机新污染物^[1]^,其种类繁多,按使用类型划分,主要包括药品与个人护理品、内分泌干扰物、农药以及阻燃剂等。虽然环境中有机新污染物的检出浓度较低,但其具有的生物累积性、内分泌干扰性及对野生动物和人体潜在的危害性,已然成为近年来备受人们关注的一大类污染物。全面了解环境中污染物的来源、分布和衰减特征对于环境污染防治和生态安全具有重要意义。《中华人民共和国国民经济和社会发展第十四个五年规划和2035年远景目标纲要》明确提出要加强新污染物治理,而检测环境中有机新污染物是治理有机新污染物的前提,只有明确有机新污染物在环境中的浓度、行为以及分布特征,才能针对性地制定治理方案。因此亟须开发实用性强的分析方法,从而灵敏、准确、高效地检测有机新污染物。

## 1 有机新污染物主要种类

### 1.1 药品与个人护理品

药品与个人护理品(pharmaceuticals and personal care products, PPCPs)是包含人用处方药和兽药在内的一类化学物质的总称,目前广泛用于生产生活的药品及个人护理用品已超过3000种^[2]^。常见的PPCPs主要包括抗生素、*β*-受体阻滞剂、抗癫痫药、血脂调节药、消炎镇痛药、抗菌药、防晒霜等^[3]^。随着人类的生产和使用,外排的PPCPs大多汇入污水处理系统,而常规的污水处理工艺如活性污泥法、生物膜法等均不能完全去除PPCPs,致使水中残留的PPCPs随出水流入受纳水体。PPCPs在水环境中的长期暴露给人类健康和水生生态环境带来潜在危害^[4,5]^,例如出现抗性基因^[6,7]^。因此近年来水环境中PPCPs的检测备受关注。

### 1.2 内分泌干扰物

内分泌干扰物(endocrine disrupting compounds, EDCs)主要包括内源性天然类固醇,如天然雌激素/雄激素、植物雌激素、真菌雌激素等和人工合成的雌激素/雄激素^[8]^以及一些工业生产所需的化工原料,如双酚A(bisphenol A, BPA)、壬基酚^[9]^和多氯联苯^[10]^等。其中,雌激素酮、雌二醇、壬基酚和BPA是地下水中检出频率最高的内分泌干扰物。已有研究^[11]^表明,EDCs会导致生物体内分泌系统紊乱,产生一系列生殖毒性,引发不可估量的生态风险。此外,开发EDCs的替代物是人们面临的另一大难题。例如,BPA及其代谢产物具有内分泌干扰效应,甚至诱发基因突变^[12]^,为了降低BPA在水环境中的风险,多种双酚类化合物被用于替代BPA,但部分替代物却具有与BPA类似甚至更强的毒性。

### 1.3 农药

农药根据用途可将分为4大类,包括用于应对植物真菌病害的杀菌剂、防治杂草的除草剂、减少虫害的杀虫剂、防治蛞蝓和蜗牛的灭螺剂^[13]^。相关研究表明,所用的农药只有5%能真正杀死害虫,其余的农药大多数进入土壤或者受纳水体^[14,15]^。亲脂性的农药通常被土壤颗粒或者有机质吸附^[16]^,而水溶性的农药则通过地表径流、污水排放和渗滤作用进入地表水和地下水^[17]^。进入环境中的农药会残留在土壤或水体中,对动物、植物、微生物以及人类健康造成严重危害^[18,19]^。

### 1.4 全氟化合物

全氟化合物是一类人工合成的化合物,通常用于制造耐油、润滑脂、耐热、耐水和耐污的产品,其分子结构为烷基链中的氢原子被氟原子饱和取代,它们的基本化学键(C-F)是最强的化学键之一。全氟化物主要包括全氟羧酸(例如全氟己酸、全氟庚酸和全氟壬酸)、全氟磺酸(例如全氟磺酰胺、*n*-乙基全氟辛烷磺酰胺乙醇和全氟己烷磺酸盐)和氟调聚物醇(如氟调聚物丙烯酸酯、氟调聚物硬脂酸酯和氟调聚物柠檬酸三酯)^[20]^。全氟化合物具有生物累积性、持久性和毒性,已在包括地表水、地下水、雨水、废水、污泥和沉积物在内的多种环境样品中检出,因此,该类物质需引起人们的重视^[21,22]^。其中,全氟辛烷磺酸和全氟辛酸是在各种环境介质中检出频率最高、研究最广泛的全氟化合物^[23,24]^。

## 2 冷冻干燥技术介绍

影响长期储存过程中物质原有特性的主要因素是水分。冷冻干燥技术是一种通过升华干燥达到除水效果的方法,其作为预处理方法具有操作简单、耗材少、样品损耗小等优点。该技术常用于农业^[25]^和食品行业^[26]^中产品的脱水与保存。制药行业^[27,28]^中药品的制备也会运用冷冻干燥技术,药品中的水分通过冷冻干燥技术处理后可得到有效的去除,产生的药品粉末能均匀地分散开,更有利于后续药品的制作。

### 2.1 冷冻干燥技术原理

冷冻干燥工艺在食品工程与制药行业的应用大体相同,通常分为3个阶段:(1)冷冻阶段;(2)初级干燥阶段;(3)再干燥阶段。

冷冻阶段是冷冻干燥的第一步,在制药和食品工程领域,水是冷冻阶段的目标。冷冻步骤中将水变成冰,使其与其他溶质成分分离。通常情况下,冷冻过程仅需几小时便能完成^[29,30]^。在此过程中,会观察到不连续的温度变化,这一现象被称为“过冷”。过冷现象的出现通常与冻结速率有关。

初级干燥阶段又称为升华干燥阶段。当腔室压力降低至冰的平衡蒸汽压以下时,物料架的温度逐步升高,产生的热量从物料架表面传递至样品。通过热传递使样品中的冰升华,随后,升华后的蒸汽转移至冷凝器中,再次转化成冰,由此,样品凝华所散失的热量将再次经由物料架传递样品,用于随后的升华过程^[31]^。初级干燥阶段是冷冻干燥3个阶段中花费时间最长的阶段。目前,有大量优化和缩短初级干燥阶段时间的研究^[32,33]^。

再干燥阶段是样品温度升高的阶段,此阶段的温度高于初级干燥阶段。样品中有部分水分在冻结阶段未变成冰,而是作为非冻结水被溶质组分捕获。在此过程中,残留的水分在样品中会发生扩散和解吸。再干燥的目的是将样品中最终残留的水分降低至可接受的水平。虽然这一阶段通常仅需几个小时,但残余的水分会使得样品质量变差,因此该阶段是冷冻干燥过程中不可或缺的一步。

### 2.2 冷冻干燥技术用于环境样品预处理的可行性

冷冻干燥技术在食品工程领域具有重要意义,通常用于食品的干燥,例如面条、面食、水果、蔬菜、虾、肉和鱼。上述食品易腐烂,作为新鲜食品难以得到良好的保存,而干燥后的食品则具有易于储存、运输成本低、包装成本低等优点,且较低的含水量不仅抑制了微生物的分解作用,还延缓了食物腐败的速度。目前,已发展出多种用于食品和药品行业的冷冻干燥技术^[34,35]^。同时,冷冻干燥广泛应用于以不稳定药物为基础的药品生产^[36]^。制药公司经常使用冷冻干燥技术延长产品的保质期,例如疫苗和其他注射剂,即通过移除产品中的水分后,将产品存储至小瓶中,便于产品的储存、运输及后期的重新定容以用于注射。以抗肿瘤药为例,冷冻干燥是一种生产稳定抗肿瘤药物的有效方法^[36]^。抗肿瘤药物的设计是一个复杂而多面性的问题,必须考虑药物的生物和药理活性、物理化学特性和剂型的生产技术。大多数抗肿瘤药物都是热不稳定且易水解,因此,在设计新制剂时,必须开发最稳定的剂型。

冷冻干燥技术在药品行业的广泛应用引人深思,在制药过程中,需保证药品的生物活性和化学活性,并维持药品的结构和功能,而这也是检测污染物的基本要求。在检测有机新污染物的样品前处理过程中,同样需保证有机新污染物结构不变,不破坏其分子。冷冻干燥技术能在制药行业中广泛应用,表明其也具备用于检测环境中有机新污染物的潜力。环境样品中的有机新污染物浓度较低,传统的样品前处理技术操作步骤繁琐,样品损失相对较大。此外,由于有机新污染物种类多且理化性质差异较大,因此容易在传统的样品前处理过程中流失,而冷冻干燥技术避免了过多的操作步骤,在浓缩样品的同时能减少样品的损失,能保证了大部分有机新污染物保存于容器中。因此,冷冻干燥技术在环境分析领域有较大的潜力。

## 3 冷冻干燥技术在环境有机新污染物分析中的应用

### 3.1 应用简介

已有少量研究将冷冻干燥技术应用于环境样品的前处理,并结合检测技术,测定环境样品中的有机新污染物,其主要的处理步骤如图1所示。目前,已有的研究主要针对抗生素和农药等有机新污染物的检测。在对样品进行冷冻干燥前,有时会对水样进行过滤^[37,38,39,40]^。针对冷冻干燥技术中的样品冷冻阶段,早期Hirsch等^[37]^和Sinha^[41]^借乙醇和甲醇达到较低的温度以快速冰冻样品,而后期Hu等^[38]^、Qiu等^[40]^和Morrison等^[39]^选择直接将样品置于-80 ℃进行冷冻。随后的干燥阶段都采用商业化的冷冻干燥机对冷冻后的环境样品进行干燥。后续步骤与传统样品前处理技术的后续步骤类似,包括以洗脱溶剂对目标污染物进行萃取、吹干、重新定容,并进行上机检测。

**图1:**
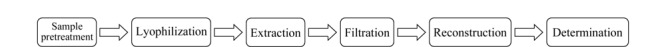
冷冻干燥技术结合检测技术处理环境样品的流程图

### 3.2 用于水环境中抗生素的检测

早在1998年,Hirsch等^[37]^就针对水环境中青霉素类、四环素类、磺胺类和大环脂类等18种抗生素,建立了冷冻干燥与高效液相色谱-质谱联用技术,并用于山泉水的检测和分析。结果表明,大部分抗生素的回收率大于80%,且相对标准偏差仅在2%~15%之间。同时,通过对比冷冻干燥和SPE两种前处理方法的效果发现,相比于冷冻干燥技术,SPE因样品体积大,浓缩倍数高,检测灵敏度更好;而冷冻干燥技术能获得更高的回收率,耗时短,更适合大量环境样品的检测。Hirsch等^[37]^使用冷冻干燥预处理水样,100 mL的样品需要经过0.45 μm的玻璃纤维滤膜过滤,加入EDTA后才能进行冷冻干燥,随后用pH 6.0的磷酸缓冲液进行定容,上机检测前用0.45 μm的聚四氟乙烯滤膜过滤以去除EDTA。虽然该方法最终获得的抗生素回收率较好,检测精度较高,但所用的冷冻干燥技术操作步骤繁琐,检测前的样品预处理步骤和过滤步骤繁多。

Hu等^[38]^结合冷冻干燥技术和LC-MS/MS,建立了一种灵敏度高、简单可靠的方法,用于检测不同类型水环境中的26种兽用抗生素,结果表明,除磺胺喹恶啉的回收率较低(只有59%)外,其他25种抗生素的回收率均高于70%,且相对标准偏差均小于20%。该方法主要是先将采集后的大体积水样(1 L)经孔径为1 μm的玻璃纤维膜过滤,再用孔径为0.45 μm的亲水性尼龙微孔滤膜过滤,以去除悬浮颗粒物,随后加入1 g柠檬酸屏蔽重金属,最终置于-20 ℃条件下避光储存。待处理时,取出部分冷冻保存的水样,解冻后充分摇匀,从中取50 mL水样进行加标,置于-80 ℃冰箱等待3 h,最后将所有样品放入冷干机冷冻干燥,干燥后的样品经有机溶剂洗脱并过滤后,进行检测。在方法建立的过程中,测试了26种抗生素在纯溶剂和水样中的稳定性,结果表明上机检测前样品应置于4 ℃条件下妥善保存,且需尽快检测。

Qiu等^[40]^将冷冻干燥技术和LC-MS/MS结合,测出了环境水样中10种氨基糖苷类的抗生素。通过分析不同加标水平的结果发现,抗生素的平均回收率在65.8%~115.7%之间,且相对标准偏差均小于20%。该方法主要将样品通过孔径为0.45 μm的膜过滤后,取10 mL水样冷冻保存6 h,而后放入冷干机冷冻干燥,干燥后的残余物用1 mL的2%乙酸洗脱并超声10 min,再经孔径为0.22 μm的尼龙膜过滤后,采用LC-MS/MS检测。该方法已用于检测50个来自广东省不同养殖场的水样,其中新霉素和链霉素在一个养猪场附近的池塘内检出量高达22.5 μg/L和36.4 μg/L。

此外,本团队^[42]^将冷冻干燥技术用于抗生素降解产物鉴定上,通过回收和浓缩微藻处理后的抗生素降解产物,利用高分辨质谱,鉴定了4种藻类降解10种抗生素的过程中产生的10种抗生素代谢产物,推测出磺胺甲恶唑、阿奇霉素、洛美沙星等抗生素在4种藻类中的转化通路。

### 3.3 用于水环境中农药的检测

Sinha等^[41]^将冷冻干燥技术引入农药的检测中,包括甲草胺、马拉硫磷、乐果、毒死蜱和赛克津。加标水平0.1 μg/L的条件下,农药的回收率在93%~104%之间,相对标准偏差低于8.62%。该方法已成功地检测出海得拉巴城市不同地区13种水样中的马拉硫磷和甲草胺。然而,该方法浓缩倍数为5,浓缩效果有限,不适用于自然水体中痕量有机新污染物,且对质谱的灵敏度要求高,因此该方法的应用存在一定的局限性。

Ramirez等^[43]^同样将冷冻干燥技术应用于检测水环境中的农药,主要目标污染物为草甘膦及其主要代谢产物氨甲基膦酸。用50 mL聚丙烯离心管采集20 mL运河水,加入天冬氨酸作为内标。为了减少冷冻干燥过程中样品的损失和交叉污染,无盖的离心管需用铝箔纸覆盖,用针扎出小孔使得冷冻干燥过程中水分升华。干燥后的残留物采用450 μL的25 mmol/L硼酸钠水溶液洗脱,并加入250 μL的500 mmol/L碱性EDTA水溶液,所得的洗脱液转入液相色谱瓶中,加入300 μL含3.75 mmol/L氯甲酸-9-芴基甲酯的乙腈,至少摇匀3 h使其充分衍生化后过滤,最终置于HPLC-荧光检测器-高效多级离子阱质谱中检测。该方法已成功检测出南佛罗里达水管理区内运河水中的草甘膦和氨甲基膦酸。

Morrison等^[39]^针对地表水中7种烟碱类农药,并结合冷冻干燥技术和LC- MS/MS,建立了定量检测方法。由于烟碱类农药几乎不被悬浮颗粒物吸附,采集的水样需先经过孔径为1.2 μm的玻璃纤维膜过滤,而后取20 mL地表水滤液加标至25 ng/L后进行冷冻,待充分冷冻后进行冷冻干燥,而后用有机溶剂洗脱残留物上的农药,待滤出液干燥后用80 μL水-甲醇-乙腈(7∶2∶1, v/v/v)重新定容,并离心过滤,最后进行检测。实验结果发现,预处理过程中添加0.1%的甲酸会降低回收率,而EDTA和柠檬酸的添加对回收率没有影响,但会增加信号的抑制作用。Morrison等^[39]^采用了添加内标抵消样品与样品间的差异,且大气压化学电离也能大幅度地减少基底效应。该方法所需样品体积小、成本和人力需求量较小,适用于较大样品量的烟碱类农药的检测。

### 3.4 用于水环境中其他有机新污染物的检测

目前对于水环境样品的前处理,冷冻干燥技术主要用来回收某一类化合物(例如抗生素和农药),而有机新污染物种类繁多,理化性质差异较大,尽可能回收多种有机新型污染物成为当前冷冻干燥技术应用于环境样品的目标之一。近期,本团队^[44]^通过冷冻干燥技术结合LC-MS/MS,成功地回收了地表水样品中38种理化性质差异较大的PPCPs,包括抗生素、心血管药物、防腐剂、脂质调节剂和非甾体消炎药抗感染药物等。大部分PPCPs的回收率处于40.0%~124.4%之间,23种PPCPs呈现低基底效应,8种PPCPs呈现中等基底效应,7种PPCPs呈现较高基底效应。除环丙沙星(29%)外,37种PPCPs的相对标准偏差低于21%。且该方法成功地用于检测水库、河流和污水处理厂出水中的PPCPs,具有实际监测水环境中有机新污染物的潜力。

冷冻干燥技术用于小体积样品(1.25 mL)检测中17*β*-雌二醇的前处理^[45]^,在此基础上,Chen等^[46]^对比了冷冻干燥、SPE和液-液萃取(liquid-liquid extraction, LLE)3种样品前处理技术,发现冷冻干燥技术与液相色谱-质谱技术联用最适宜分析17*β*-雌二醇和雌酮,只需将50 mL的水样冷冻干燥后,用丙酮洗脱后吹干,以0.5 mL乙腈-水(1∶1, v/v)定容,0.45 μm孔径的小型非针式滤器(Whatman,英国)过滤后,置于LC-MS/MS中检测。

### 3.5 冷冻干燥技术与其他技术的对比

不同环境样品前处理方法各有优缺点(见表1),现有的样品前处理技术主要包括超声辅助萃取(ultrasound-assisted extraction, USE)^[47]^、QuEChERS(quick, easy, cheap, efficient, rugged, and safe)^[48]^、LLE^[49]^、固相萃取(solid-phase extraction, SPE)^[50]^、固相微萃取(solid phase microextraction, SPME)^[51]^和搅拌棒固相萃取(stir bar sorptive extraction, SBSE)^[52]^等。冷冻干燥技术虽无需耗材(如较昂贵的SPE小柱、SPME萃取头或SBSE搅拌棒),有机溶剂消耗少,但与SPE、SPME和SBSE相比样品浓缩倍数小,浓度较低的化合物可能较难测出。在环境样品的前处理过程中,冷冻干燥技术的净化效果有限,环境样品基底会对污染物的检测产生干扰,因此冷冻干燥技术适宜较洁净样品的前处理,例如饮用水、水源水、地下水和洁净的地表水等。

**表1:** 不同预处理方法处理环境样品的比较

Pretreatment method	Sample amount/mL	Organic solution/mL	Concentration factor	Equilibrium time/min	Consumables	Reference
USE	5.00^*^	11	11	no need	no need	[47]
QuEChERS	2.00^*^	11	10	no need	no need	[48]
LLE	100	not mention	not mention	overnight	no need	[49]
SPE	1000	21	1000	no need	HLB cartridges	[50]
SPME	10	0.020	500	40	PDMS-DVB fiber	[51]
SBSE	50	2.1	250	230	PDMS stir bar	[52]
Lyophilization	50	1	50	no need	no need	[38]

* Sample mass, g.

## 4 展望

综上所述,冷冻干燥技术具有成本低、操作简单、耗材少、有机溶剂消耗少、水样处理体积小、样品可保存及处理过程中目标物损失少等优点。其中,冷冻干燥技术成本低的优势,为环境样品中有机新污染物的开展提供有利条件,使得人们能更全面深入的了解有机新污染物的环境行为。大多数有机新污染物在自然环境中的浓度处于ng/L的水平,预处理过程中样品损失对最终的检测影响较大,当样品量较少时,避免样品的损失尤为重要,而冷冻干燥技术避免样品损失的优势,使其替代部分前处理技术成为可能。已有部分研究将冷冻干燥技术成功用于环境样品中有机新污染物的预处理。冷冻干燥技术的发展趋势如图2所示。值得关注的是,大多数研究者主要关注某一类有机新污染物的检测,如抗生素和农药,未来可将冷冻干燥技术用于更多类别的有机新污染物的预处理。目前还未发现将冷冻干燥技术用于水环境中有机新污染物的例行性和周期性监测,未来可考虑用冷冻干燥技术代替部分SPE等前处理工作,用于更大规模的高频采样中,更加细致地监测有机新污染物的环境行为,为管控有机新污染物的排放提供信息。此外,未来还可结合冷冻干燥技术和全扫描质谱,实现快速筛选环境样品中的未知有机新污染物。

**图2:**
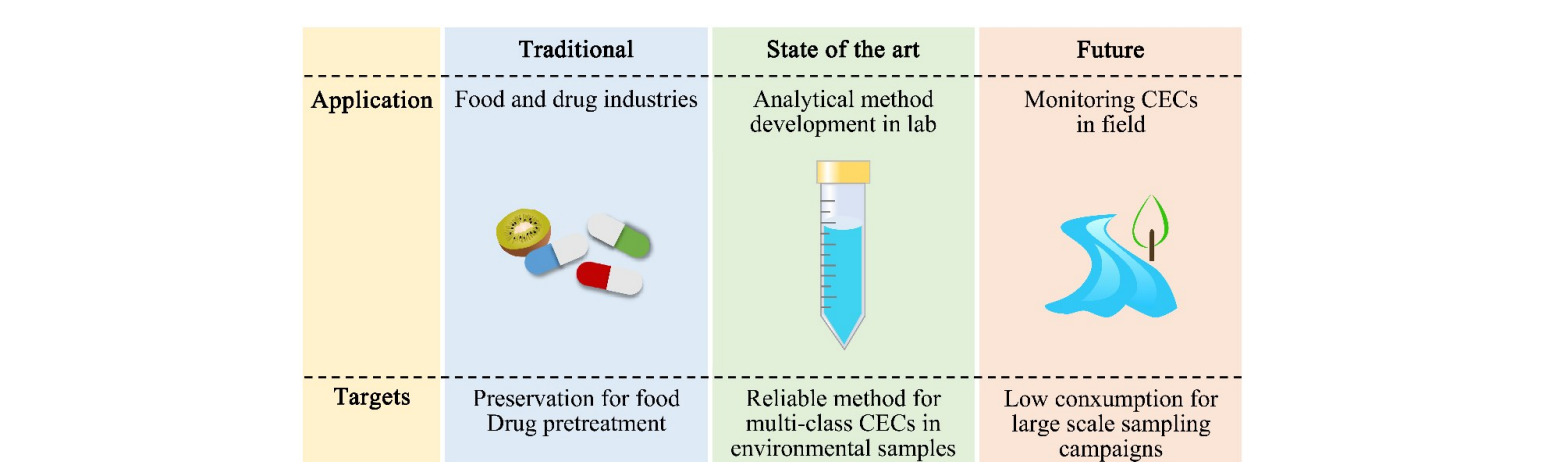
冷冻干燥应用的过去、现在和未来
